# IgG Anti-Spike Antibodies and Surrogate Neutralizing Antibody Levels Decline Faster 3 to 10 Months After BNT162b2 Vaccination Than After SARS-CoV-2 Infection in Healthcare Workers

**DOI:** 10.3389/fimmu.2022.909910

**Published:** 2022-06-15

**Authors:** Bram Decru, Jan Van Elslande, Sophie Steels, Gijs Van Pottelbergh, Lode Godderis, Bram Van Holm, Xavier Bossuyt, Johan Van Weyenbergh, Piet Maes, Pieter Vermeersch

**Affiliations:** ^1^ University Hospitals Leuven, Clinical Department of Laboratory Medicine and National Reference Center for Respiratory Pathogens, Leuven, Belgium; ^2^ Academic Centre of General Practice, KU Leuven, Leuven, Belgium; ^3^ Department of Public Health and Primary Care, KU Leuven, Leuven, Belgium; ^4^ Environment and Health, Department of Public Health and Primary Care, KU Leuven, Leuven, Belgium; ^5^ Group IDEWE, External Service for Prevention and Protection at Work, Heverlee, Belgium; ^6^ Laboratory of Clinical and Epidemiological Virology, Department of Microbiology, Immunology and Transplantation, Rega Institute, KU Leuven, Leuven, Belgium; ^7^ Department of Cardiovascular Sciences, KU Leuven, Leuven, Belgium

**Keywords:** SARS-CoV-2, COVD-19 serological testing, neutralizing antibodies, vaccination, spike, IgG, immunity, COVID-19

## Abstract

**Background:**

IgG anti-spike (S) antibodies arise after SARS-CoV-2 infection as well as vaccination. Levels of IgG anti-S are linked to neutralizing antibody titers and protection against (re)infection.

**Methods:**

We measured IgG anti-S and surrogate neutralizing antibody kinetics against Wild Type (WT) and 4 Variants of Concern (VOC) in health care workers (HCW) 3 and 10 months after natural infection (“infection”, n=83) or vaccination (2 doses of BNT162b2) with (“hybrid immunity”, n=17) or without prior SARS-CoV-2 infection (“vaccination”, n=97).

**Results:**

The humoral immune response in the “vaccination” cohort was higher at 3 months, but lower at 10 months, compared to the “infection” cohort due to a faster decline. The “hybrid immunity” cohort had the highest antibody levels at 3 and 10 months with a slower decline compared to the “vaccination” cohort. Surrogate neutralizing antibody levels (expressed as %inhibition of ACE-2 binding) showed a linear relation with log10 of IgG anti-S against WT and four VOC. IgG anti-S corresponding to 90% inhibition ranged from 489 BAU/mL for WT to 1756 BAU/mL for Beta variant. Broad pseudoneutralization predicted live virus neutralization of Omicron BA.1 in 20 randomly selected high titer samples.

**Conclusions:**

Hybrid immunity resulted in the strongest humoral immune response. Antibodies induced by natural infection decreased more slowly than after vaccination, resulting in higher antibody levels at 10 months compared to vaccinated HCW without prior infection. There was a linear relationship between surrogate neutralizing activity and log10 IgG anti-S for WT and 4 VOC, although some VOC showed reduced sensitivity to pseudoneutralization.

## Introduction

Vaccines are an important weapon in the fight against the COVID-19 pandemic. The BNT162b2 mRNA (Pfizer) is one of the most used vaccines worldwide and proved highly effective in clinical trials as well in real-life epidemiological studies that included millions of study subjects (1, 2). This vaccine elicits a strong antibody response against the spike (S) antigen, but not against the nucleocapsid (N) antigen. Anti-N antibodies can therefore be used as a surrogate marker for natural infection after vaccination with BNT162b2. Vaccine effectiveness studies have shown a decline of protection against symptomatic infection over time, while protection against severe disease is better retained (3–5). The waning of vaccine-induced immunity has been attributed to declining anti-S antibody levels and the emergence of variants of concern (VOC). Anti-S antibody levels start to decline 2-3 months after vaccination, declining continuously up to 8 months after vaccination (6–8). A similar continuous decline has been observed for anti-S and anti-N antibodies after natural infection (9, 10).

The receptor binding domain (RBD) on the S antigen is the target of most neutralizing antibodies (Nabs), as RBD mediates viral entry through binding of the ACE2-receptor on human cells. It has been well demonstrated that the level of Nabs correlates with protective immunity (3, 11–13). Live-virus plaque reduction neutralization tests (PRNT), considered the gold standard for measuring Nabs, are cumbersome, expensive and require a biosafety level (BSL) 3 laboratory environment. Furthermore, these assays have suboptimal interlaboratory reproducibility as there is no standardized protocol that is universally used (14–16). As an alternative to PRNT, the correlation of antibodies directed against the spike RBD measured with routine laboratory assays (“binding” antibodies) could in theory be used to infer protection (12). These tests are routinely available, easier and less expensive to perform (17). As the correlation of the results of “binding” antibody assays with live-virus neutralization is not excellent, assay-dependent and changes over time after infection or vaccination, an exact Nab titer cannot be predicted based on the level of “binding” antibodies (7, 18). Measuring surrogate neutralizing antibodies (pseudoneutralization) is an intermediate way between binding antibody tests and live-virus Nab tests, giving a better approximation of Nab titers and better reproducibility, while still suitable to be performed in a routine laboratory environment (BSL-2) (15, 16).

## Materials and Methods

### Study Design

This study was approved by the local ethics committee at the University Hospitals Leuven (S64152). After obtaining informed consent, serum samples were collected from a cohort of health care workers (HCW) who received two doses of the BN162b2 vaccine (with a 21-day interval (20-22 days). The results of the first 4 time points up to 3 months after the first dose have been previously published (19). For this follow-up study, only participants who were sampled at both 3 and 10 months were included (114 of the 150 participants).

The study cohort included 97 participants who were COVID-19 naive when vaccinated (“vaccination” cohort) and 17 participants who had a SARS-CoV-2 infection 6 to 10 months prior to vaccination (“hybrid immunity” cohort). Infection in the 17 participants with “hybrid immunity” was confirmed by PCR or serological evidence of previous infection at baseline (positive for IgG anti-S and anti-N antibodies). Five participants in the “vaccination” cohort and 1 participant in the hybrid immunity cohort had a breakthrough infection between the 3 and 10-month time points (5 confirmed by PCR and 1 by a 3-fold or higher increase in IgG anti-S and anti-N titer). In addition, 17 participants in the “vaccination” cohort had already received a booster dose of BN162b2 before sample collection at 10 months (16 participants between 2-7 weeks and one participant 5 days before sampling). These 23 participants were excluded from all statistical analyses.

Antibody kinetics after vaccination were compared to antibody kinetics after natural infection in a cohort of HCW (“infection” cohort). This cohort consisted of HCW included in a prior arm of the same clinical study (S64152) which studied Abbott IgG anti-S and anti-N levels in HCW up to 10 months after natural infection (9). The criterion for inclusion in the current study was the availability of a sample collected at 3 months (80-100 days, median 87 days, n= 83) and a sample collected at 10 months (280-320 days, median 295 days, n=83) after positive PCR. These participants were first sampled in June-July 2020 and a second time in December 2020-January 2021, before vaccines were available for HCW in Belgium. These natural infections (March to June 2020) precede the emergence of VOC and thus correspond to “Wild Type (WT)” strains. There were no reinfections between 3 and 10 months in the infection cohort. Samples used for surrogate neutralizing antibody measurement were stored at -20°C before analysis.

### Patient Population

The patient characteristics of the three cohorts are described in **Table 1**. The median interval in the “hybrid immunity” cohort between positive PCR and administration of the first dose of vaccine was 274 days (range 39-301 days).

**Table 1:** Patient demographics.

	“Vaccination” cohort	Hybrid immunity^1^	“Infection” cohort
Number of individuals	97	17	83
Age (SD)	50.2 (13.8)	52.2 (9.3)	46.6 (11.8)
Female/male (%female)	67/30 (69.1)	16/1 (94.1)	72/11 (86.7)
Severity (WHO score)			
*0*	97 (100.0)	4 (23.5)	7 (8.4)
* 1*		9 (52.9)	70 (84.3)
* 2*		4 (23.5)	6 (7.2)
Individuals			
* 3 months*	97	17	83
* 10 months*	97	17	83
Days after vaccination/positive PCR			
* at 3 months (SD)*	91.17 (1.38)	91.59 (3.86)	88.16 (5.21)
* at 10 months (SD)*	296.3 (6.2)	296.8 (6.0)	293.8 (6.7)
New SARS-CoV-2 exposure between 3 and 10 months?²			
* no new exposure*	75 (77.3)	16 (94.1)	83 (100.0)
* breakthrough infection*	5 (5.2)	1 (5.9)	
* booster vaccination*	17 (17.5)		

**^1^** Median time between positive PCR and administration of the first dose was 274 days (range:39-301).

**^2^** Individuals who had a booster or breakthrough infection were excluded for the kinetics analyses of binding and surrogate neutralizing antibodies.

### Antibody Measurement

IgG antibodies against SARS-CoV-2 nucleocapsid and spike RBD were measured with the Abbott Architect (Abbott, Lake Forest Illinois) SARS-CoV-2 IgG (anti-N) and IgG II Quant (anti-S) chemiluminescence immunoassays using the manufacturer’s cut-offs for positivity of 1.4 S/CO and 50 AU/mL, respectively. IgG anti-N was only measured at baseline and at 10 months. Samples above the measuring range of 40.000 AU/mL for anti-S were further diluted 1:2 using Abbott multi-assay manual diluent according to the manufacturers’ instructions. The units of the quantitative Abbott anti-S assay (AU/mL), which uses a 6-point calibration curve, were converted to WHO units (BAU/mL) by multiplying with a factor of 0.142 according to the manufacturer’s instructions. This is not possible for the semi-quantitative Abbott anti-N assay (S/CO) which only uses a two-point calibration curve.

### Surrogate Neutralizing Antibodies

Detection of surrogate neutralizing antibodies was performed using the V-PLEX ACE2 neutralization assay panel 13 (K15466U, Meso Scale Diagnostics, LCC. Rockville, USA). This panel is an extension of panel two, which was chosen by Operation Warp Speed as the basis of its standard binding assays for immunogenicity assessments in all funded Phase III clinical trials of vaccines (20). It is a competitive binding assay that quantitatively measures antibodies that block the binding of ACE2 of its cognate ligands. Neutralizing capacity is quantified by measuring light emitted from a conjugated ACE2 protein which binds non-blocked spike antigens. The more neutralizing antibodies in the patient sample, the more the binding of ACE2 to the coated spike proteins is inhibited (results are expressed in %inhibition). Samples were prediluted 1/10 according to the manufacturer’s instructions. The assay contains coated SARS-CoV-2 spike proteins of the following variants: Wild Type (Wuhan strain), Alpha (B.1.1.7), Beta (B.1.351), B.1.526.1, B.1.617, Kappa (B.1.617.1), Delta (B.1.617.2), B.1.617.3, Gamma (P.1) and zeta (P.2). We only included the WT strain and the four VOC included in the panel (Alpha, Beta, Delta and Gamma strains) for further analysis. Comparable surrogate neutralization assays have been shown to correlate well with conventional live virus neutralization assays (r = 0.93) (16).

### Omicron BA.1 Virus Neutralization

Twenty serum samples with >90% inhibition at 1/10 dilution with the V-PLEX ACE2 neutralization assay against Wild Type variant (94.8 ± 1.8%) were randomly selected for live virus (Omicron BA.1 VOC) neutralization on Vero cells, as previously described (21). Live virus neutralization was performed with serial twofold dilutions of serum (1/50 to 1/1600) and results were expressed as % inhibition at 1/100 dilution or 50% Neutralizing Titer (NT50) of cytopathogenic effect.

### Data Analysis

The median antibody levels (three cohorts) and %inhibition (three cohorts and 5 variants) were compared using the non-parametric rank Mann–Whitney U (MWU) test with R Studio and corrected for multiple testing using the Bonferroni method. Decline rates are reported as the median value within a cohort of individual decline rates. A p-value <0.05 was considered significant.

The log10 of the antibody titers and the log10 decline in antibody titers between 3 and 10 months were compared using the non-parametric Mann-Whitney-U test. The decline of antibody titers between 3 and 10 months was calculated by performing a simple linear regression in R studio using the log10 of the antibody titer and days post positive RT-PCR, corresponding to a one-phase exponential decay of the antibody levels.

Simple linear regression was used to calculate antibody levels (BAU/mL) correlating to 50%, 90% and 95% surrogate neutralizing inhibition levels. Spearman correlation was used to evaluate the relationship between the log10 binding antibody titer, and surrogate neutralizing inhibition levels (%), and live virus neutralization.

Surrogate neutralizing antibodies against WT and 4 VOC of the 20 samples selected for live virus neutralization were compared with One-way ANOVA with stringent FDR correction for multiple comparisons.

## Results

### IgG Anti-S Antibody Kinetics

**Figure 1:**
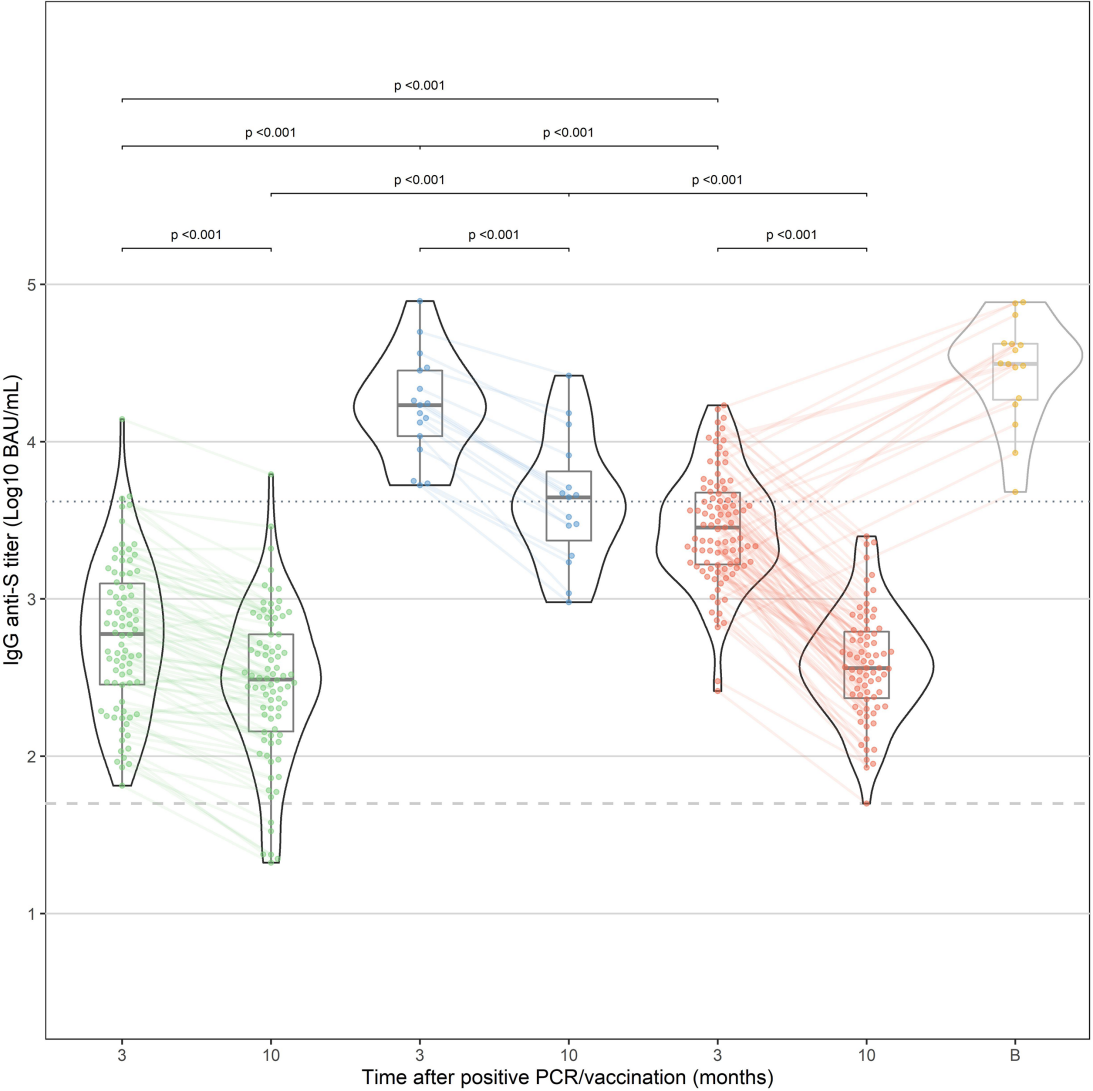
IgG anti-S antibody response after natural infection (“infection”, green) and after vaccination in previously infected HCW (“hybrid immunity”, blue) and naive HCW (“vaccination”, red). In addition, the results at 10 months of HCW who have received a booster dose before the 10-month time point are shown in yellow (excluded for statistical analysis). Median time after booster (shown as time point "B") was 21 days (IQR: 13.8-39.5 days).

**Figure 2:**
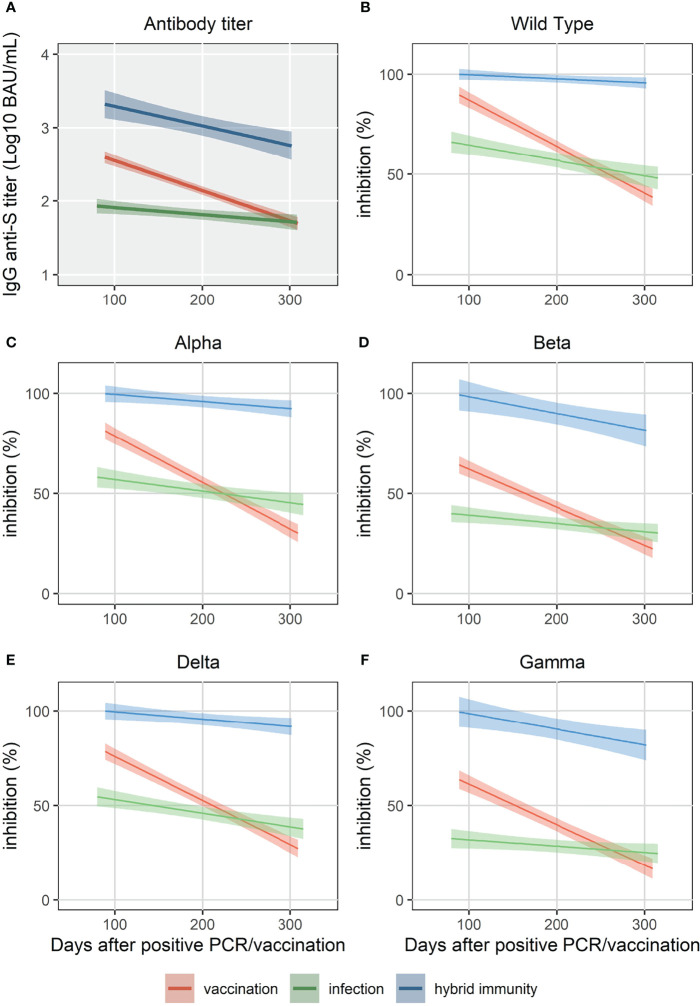
Simple linear regression of IgG anti-S **(A)** and surrogate neutralizing antibody levels (% inhibition) for WT and 4 VOC **(B-F)** up to 320 days.

All HCW in the “vaccination” and “infection” cohorts had detectable IgG anti-S antibodies within 3 months after vaccination. After 10 months, all vaccinated HCW were still seropositive compared to 92.7% of the “infection” cohort. IgG antibody levels in the “hybrid immunity” cohort were significantly higher at 3 and 10 months than in the “vaccination” and “infection” cohorts (p<0.001 for both time points, **Figure 1**). After 10 months, median decline of antibody levels compared to 3 months was 68%(p<0.001), 87% (p<0.001) and 49% (p<0.001) in the “hybrid immunity”, “vaccination”, and “infection” cohort, respectively. The relative decline of antibody levels was significantly faster in the “vaccination” cohort compared to the “infection” cohort and the “hybrid immunity” cohort (p<0.001 *vs*. both with MWU test). **Figure 2A** shows a simple linear regression of log10 IgG anti-S Antibody decline between 3 and 10 months in the three groups.

### Surrogate Neutralizing Antibody Kinetics Against Wild-Type and Four VOC

For the “vaccination” and “infection” cohort, %inhibition was significantly higher at both 3 and 10 months against the WT strain compared to Beta, Gamma and Delta VOC (p<0.05), but not compared to Alpha VOC. For the “hybrid immunity” cohort, we did not find a significant difference between WT and the four VOC at 3 and 10 months as %inhibition was close to 100% for all samples.

The “hybrid immunity” cohort showed the highest %inhibition at 3 and 10 months against all the strains compared to the “vaccination” and “infection” cohorts (p<0.001 *vs*. both for all strains). Median %inhibition in the “hybrid immunity “cohort was 100% for WT and the four VOC at 3 months. At 10 months, median %inhibition was 100% for WT, and between 95% (Beta) and 99% (Delta) for the four VOC.

**Figure 3:**
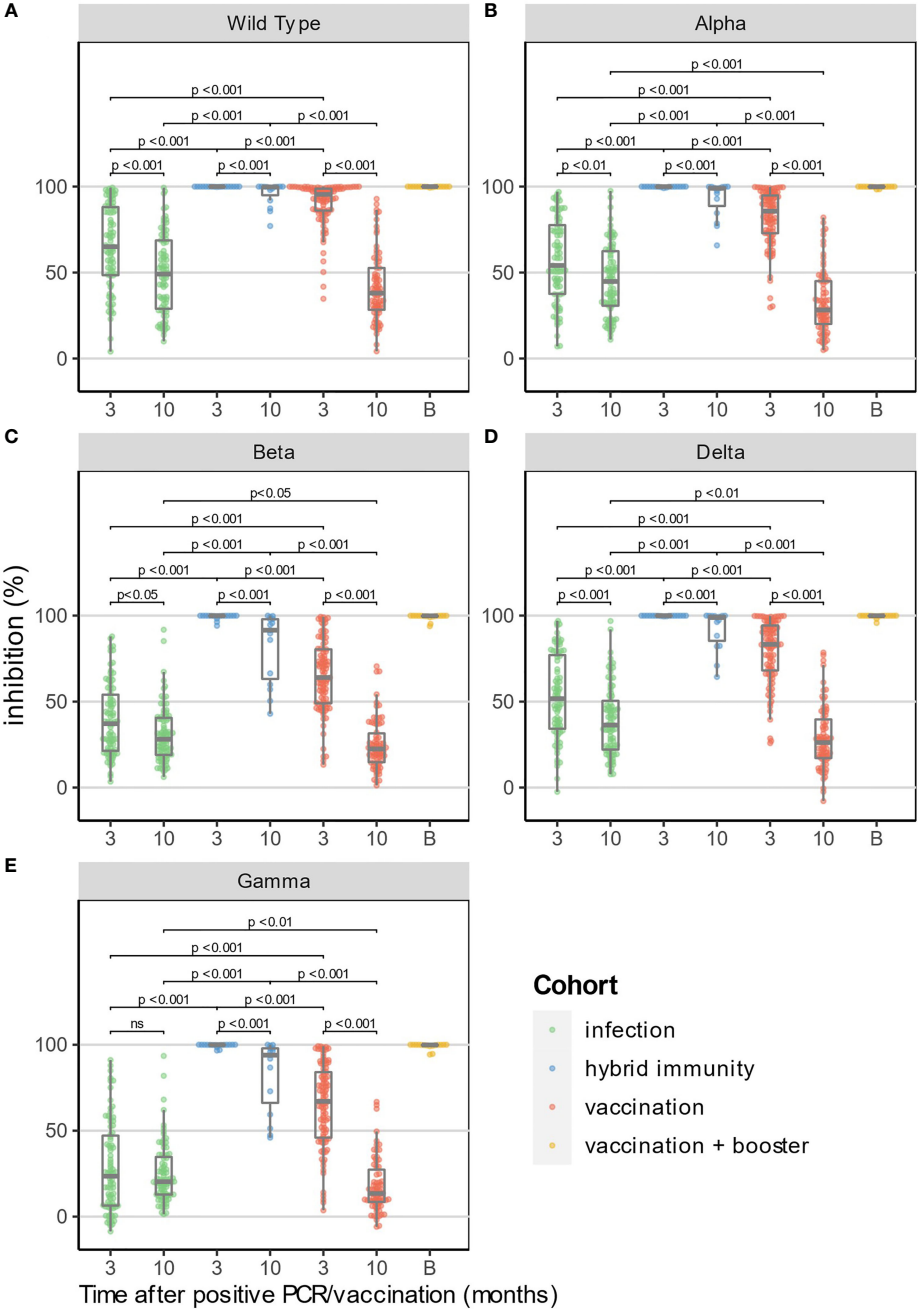
Surrogate neutralizing antibody levels (% inhibition) at 3 and 10 months for WT and 4 VOC **(A–E)**. In addition, the results at 10 months of HCW who have received a booster dose before the 10-month time point are shown in yellow (excluded for statistical analysis). Median time after booster (shown as time point "B") was 21 days (IQR: 13.8-39.5 days).

At 3 months, %inhibition for WT and the four VOC was significantly lower in the “infection” cohort than in the “vaccination” cohort (p<0.001 for all strains, **Figure 2**). Ten months after vaccination, the median %inhibition decrease was 51% for WT and between 40% (Beta) and 52% (Delta) for the four VOC in the “vaccination” cohort. In the “infection” cohort, however, the median %inhibition decrease was only 16% for WT and between 7% (Beta) and 14% (Delta) for the four VOC. Due to the faster relative decrease in %inhibition in the “vaccination” cohort (p<0.001 for each strain *vs*. “infection” cohort, MWU-test), median %inhibition at 10 months in the “vaccination” cohort was significantly lower compared to the “infection” cohort for the four VOC (p<0.05), but not for WT (p=0.08) (**Figures 3A-E**).

### Correlation Between IgG Anti-S Antibody and Surrogate Neutralizing Antibody Levels

**Figure 4:**
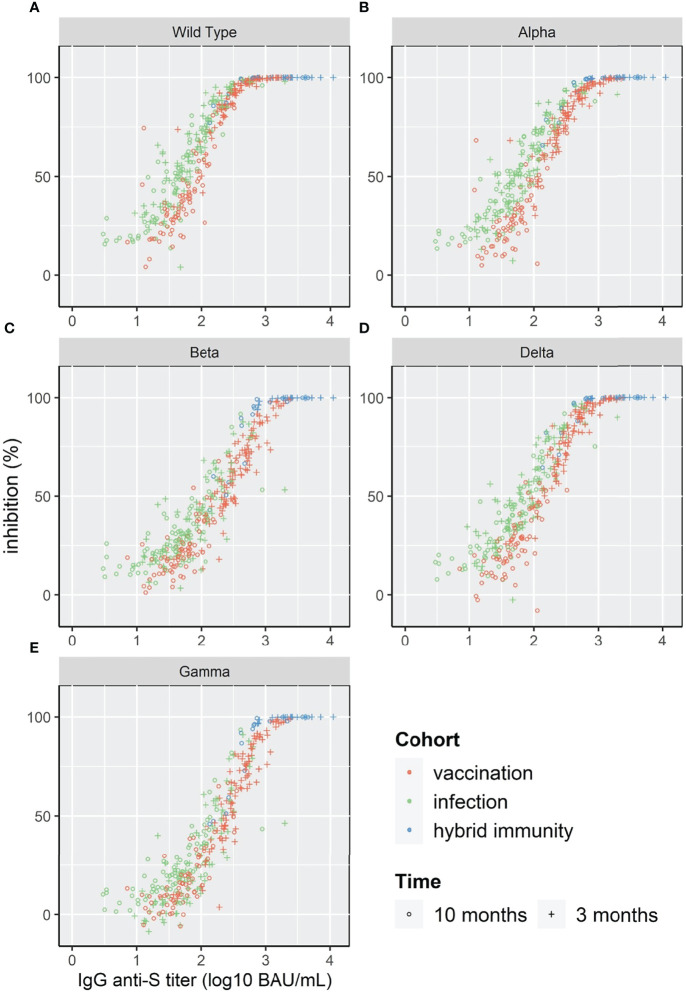
Scatterplot of surrogate neutralizing antibody inhibition levels (%) and IgG anti-S for WT and 4 VOC **(A–E)**.

The log10 of IgG anti-S showed a linear relation with %inhibition reaching a plateau close to 100% inhibition for WT and the four VOC (**Figure 4**). Given the relatively low number of individuals in our study, the three cohorts were pooled for correlation analysis. The Spearman’s rank correlation coefficient was significantly higher at 3 months than at 10 months for the four VOC and the Wild Type strain (**Table 2**).

**Table 2:** Spearman rank correlation of %inhibition for WT and the four VOC with IgG anti-S.

Time	Variant	Rho (95% CI interval)	p-value
**3 months**	Wild Type	0,97 (0,96-0,98)	9,2E-102
**3 months**	Alpha	0,95 (0,94-0,97)	3,2E-88
**3 months**	Beta	0,92 (0,9-0,94)	2,1E-70
**3 months**	Delta	0,95 (0,94-0,97)	4,9E-88
**3 months**	Gamma	0,94 (0,93-0,96)	3,4E-82
**10 months**	Wild Type	0,86 (0,81-0,89)	2,6E-49
**10 months**	Alpha	0,8 (0,74-0,85)	2,5E-38
**10 months**	Beta	0,79 (0,72-0,84)	1,0E-35
**10 months**	Delta	0,83 (0,78-0,87)	1,4E-43
**10 months**	Gamma	0,8 (0,74-0,85)	8,2E-38

To compare the correlation at different antibody levels for the different strains, we calculated the IgG anti-S titer that corresponded to 50%, 90% and 95% inhibition (**Table 3**). The IgG anti-S titer corresponding to 90% inhibition was 489 BAU/mL for WT with an agreement of 89% (above/below cut-off) and varied between 713 BAU/mL (Alpha) and 1756 BAU/mL (Beta) for the four VOC with an agreement of ≥92%.

**Table 3:** Calculated IgG anti-S titer corresponding to 50%, 90% and 95% inhibition and % agreement (%AG, above below cut-off for antibody titer and %inhibition) for the different strains.

	50% Inhibition		90% Inhibition		95% Inhibition	
	BAU/mL	%AG	BAU/mL	%AG	BAU/mL	%AG
**Wild Type**	48	*87%*	489	*89%*	653	*90%*
**Alpha B.1.1.7.**	70	*87%*	713	*92%*	954	*94%*
**Beta B.1.351.**	158	*91%*	1756	*94%*	2373	*95%*
**Delta B.1.617.2.**	90	*91%*	814	*92%*	1071	*93%*
**Gamma P.1.**	203	*91%*	1626	*93%*	2109	*95%*

### High Titers of Pseudoneutralization Against Alpha, Beta, Gamma and Delta VOC Predict Neutralization of Omicron BA.1

We investigated if high pseudoneutralization values against Alpha, Beta, Gamma and Delta VOC, in serum samples collected before the arrival of the Omicron variant, might predict the presence of neutralizing antibodies against the Omicron BA.1 variant in a live virus-neutralization assay in Vero cells, as previously described (21).

**Figure 5:**
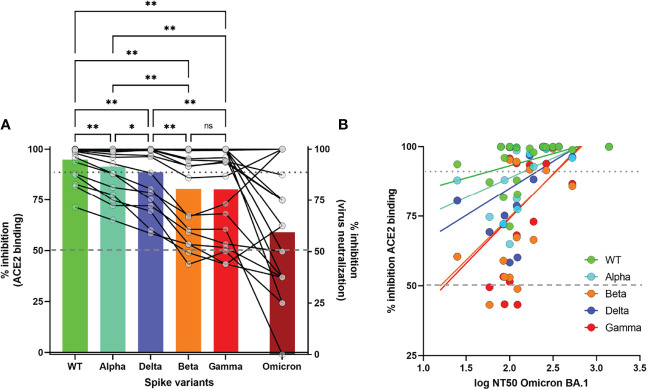
Correlation of surrogate neutralizing antibody levels (%inhibition) against WT and 4 VOC with virus-neutralization assay for Omicron BA.Pseudoneutralization is expressed as % inhibition of recombinant ACE2 binding for 4 VOC. Virus neutralization of Omicron is expressed as % inhibition at 1/100 dilution (**Figure 5(A)**) or 50% Neutralizing Titer (NT50) of cytopathogenic effect (**Figure 5(B)**). Statistical analysis by One-way ANOVA with stringent FDR correction for multiple comparisons (**p<0.01, *p<0.05, ns, not significant). Correlation between both assays was measured by Spearman’s Rho coefficient (p<0.05 for all 5 Spike variants). Dashed and dotted grey lines depict 50% and 90% inhibition, respectively.

Of the 20 randomly selected serum samples with >90% inhibition (94.8% ± 1.8%) against WT pseudovirus, 4/20 (20%) displayed 100% Omicron neutralization at 1/100 dilution, while 13/20 (65%) showed at least 50% neutralization against Omicron (**Figure 5A**). We observed a gradual decrease in (pseudo)neutralization across all VOC as compared to WT (Alpha<Delta<Beta/Gamma<Omicron), in agreement with the extensively described antigenic distance among these VOC (22). Pseudoneutralization of all 5 Spike variants (1/100 dilution) was significantly correlated with Omicron BA.1 virus neutralization (**Figure 5B**, all p<0.05). However, a high %inhibition against Beta and Gamma VOC was the strongest predictor of Omicron neutralization, with a cutoff of 70% inhibition resulting in 84.6% sensitivity and 71.4% specificity of predicting high (>50%) Omicron neutralization (RR 2.96 95%CI[1.21-10.44], Fisher’s exact test p=0.022).

## Discussion

We studied the IgG anti-S and surrogate neutralizing antibody kinetics (expressed in %inhibition of ACE-2 binding), after vaccination with BNT162b2 and natural infection up to 10 months. Vaccination after prior infection (“hybrid immunity”) resulted in the highest IgG anti-S antibody levels and %inhibition and the slowest decline between 3 and 10 months. Vaccination in COVID-19 naive individuals resulted in higher IgG anti-S antibody titers and %inhibition for WT and four VOC at 3 months compared to natural infection, but not at 10 months due to a faster decline after vaccination. IgG anti-S correlated well with neutralizing activity againstWT and 4 VOC at 3 and 10 months. High titers of pseudoneutralization at 10 months (vaccinated with 2 doses BNT162b2) predicted live virus neutralization of Omicron BA.1.

IgG anti-S titers ("binding antibodies") after natural infection and vaccination correlate with (pseudo)neutralizing titers (23, 24), and neutralizing titers, in turn, correlate with protection against disease (3, 11–13). Therefore, the kinetics of IgG anti-S "binding antibodies", neutralizing antibodies and protective immunity often show a similar course in studies (1, 3, 7, 11, 12) and protection can be estimated using IgG anti-S titers (12). The exact degree of correlation depends on the assays used (e.g. total Ig *vs* IgG) and the threshold of protection depends on disease severity and the SARS-CoV-2 variant (3, 11, 25). Despite waning over time, residual humoral immunity may still be sufficient to protect against severe disease as lower amounts of neutralizing antibodies are required for protection against severe disease compared to mild infection (1, 3, 5, 11, 26).

We found a linear relationship between the log10 of binding IgG anti-S levels and surrogate neutralizing antibodies for WT and four VOC. Using a 1:10 dilution of the serum, 90% inhibition corresponded to IgG anti-S titers between 489 BAU/mL for WT and 1756 BAU/mL for the Beta variant. Although the shape of the relation between binding antibodies and %inhibition was similar for all variants, the level of IgG anti-S antibodies in BAU/mL required to reach 90% inhibition varied. This implies that the prediction of protective immunity is dependent on the circulating variant. When a new variant arises (such as the recent Omicron), the relationship between IgG anti-S antibodies and neutralizing capacity must be reassessed. In addition, the correlation also depends on the time after infection/vaccination. Similar to Levin et al., we found a lower Spearman’s rank correlation for WT and the four VOC at 10 months compared to at 3 months (7). Nonetheless, the correlation at 10 months was still good (ρ ≥0.79).

Waning of antibody levels after vaccination, with concomitant reductions in protective immunity, have been demonstrated in clinical trials as well as in epidemiological studies (4, 7, 27–29). In HCW, IgG anti-S antibody levels peak around 3 weeks after the second dose of BNT162b2, followed by a decline over time (19). In the phase 3 trial of BNT162b2, vaccine protection peaked between 7 days and 2 months after the second dose, followed by a progressive decline of protection (84% in the period 4-7 months after the second dose) (1). Andrews et al. showed a reduction of vaccine effectiveness of BNT162b2 of 96% early after vaccination to 66.3% at 5 months after the second dose (against the Delta variant) (4). Sheehan et al. showed immunity after vaccination with BTN162b2 already drops to around 50% after only 6 months (30).

In contrast, protection induced by “natural” infection seems to be better preserved over time, with protection rates of 85-95% against symptomatic infection up to one year after infection, even against VOC such as the Beta variant (30–32). The decline of antibody titers is also reported to be slower after natural infection compared to vaccination up to 6 months (33). Our findings of high antibody levels and surrogate neutralizing titers early after vaccination, but faster decline compared to naturally induced immunity, corroborate and extend the findings of these studies up to 10 months and against four VOC. We confirmed lower neutralizing activity against 3 VOC that contain spike mutations conferring immune escape such as the E484K (Beta and Gamma) and the L452R and T478K (Delta) (4, 34–36). The Beta and Gamma VOC show the greatest reduction in %inhibition compared to WT, corresponding to the greatest immune escape, while intermediary levels were noted for the Delta variant. High pseudoneutralization values against WT and 4 VOC at 10 months predicted the presence of neutralizing antibodies against the Omicron BA.1 with 17/20 (85%) of the samples with >90% inhibition against WT showing at least 50% neutralization against Omicron in live virus neutralization at a 1/100 dilution. High surrogate neutrolizing antibody titers against Beta and Gamma VOC, the two VOC with the highest immune escape, were the strongest predictors of Omicron neutralization.

Individuals with hybrid immunity have been shown to exhibit the highest (neutralizing) antibody levels and the best protection against reinfection (8, 37–39). This is already apparent in the first weeks after vaccination and persists up to 8 months (8, 37–39). Individuals with hybrid immunity have higher peak antibody levels and a slower decline over time up to 10 months (33, 33, 40). Our results confirm higher antibody levels and a slower decline up to 10 months in HCW with hybrid immunity. In addition, we showed that surrogate neutralizing activity was highest in the hybrid immunity cohort both at 3 and 10 months.

Taken together, our data support the administration of a booster dose within one year after initial vaccination with two doses of BNT162b2, especially in COVID-19 naive individuals where antibody levels and neutralizing capacity have waned significantly by 10 months. Booster doses have been shown to increase (neutralizing) antibody titers and concomitant protection levels, providing the rationale for the widespread booster program rollout around the world (41). The approach is further supported by the fact that immunity after vaccination with 2 doses of BNT162b2 against the recent Omicron variant showed a similar course with a peak around 65.5%, dropping to only 8.8% after more than 25 weeks in COVID-19 naive individuals while protection against reinfection was still 56.0% over 300 days after natural infection (26, 42).

A strength of our study is the use of three cohorts of HCW with comparable demographics with follow-up up to 10 months. There are also a few limitations to our study. A first limitation is the relatively small number of participants. We believe that the same participants were sampled at different time points and the comparable demographics of the different cohorts at least in part offset this limitation. A second limitation is the lack of a live-virus PRNT for WT and 4 VOC which is the golden standard technique for analyzing the neutralizing activity of serum. Nevertheless, a good correlation between pseudoneutralization and PRNT has previously been demonstrated and is supported by our data (15, 16). Finally, we did not assess cellular components of adaptive immunity which might play an important role in the persistence of protection against (severe) disease over time (43).

## Conclusion

Hybrid immunity resulted in the strongest immune response at 3 and 10 months for WT and four VOC. Vaccination in COVID-19 naive HCW resulted in a stronger humoral immune response compared to natural infection at 3 months, but not at 10 months. This was due to a faster decline of IgG anti-S and surrogate neutralizing antibody levels after vaccination in COVID-19-naive HCW, compared to natural infection. There was linear relationship between log10 of binding IgG anti-S and surrogate neutralizing antibodies for WT and four VOC, although the binding antibody level corresponding to 90% inhibition varied depending on the strain.

## Data Availability Statement

The raw data supporting the conclusions of this article will be made available by the authors, without undue reservation.

## Ethics Statement

The studies involving human participants were reviewed and approved by The Ethics Committee Research (EC Research) of University Hospitals Leuven (UZ Leuven). The patients/participants provided their written informed consent to participate in this study.

## Author Contributions

Conceptualization: JE and PV; Methodology: PV, JE, JW, and BD; Formal analysis: BD and JW; Investigation: BD, JW, BH, and SS; Resources: GP and LG; Data curation: BD, JW, PM, BH, and JE; Writing – original draft preparation: JE, BD, and PV; Writing – review and editing: JE, BD, PV, SS, GP, LG, XB, JW, and PM; Visualization: BD, JW, and JE; Supervision: PV, PM, and JW; Project administration: PV; Funding acquisition: PV. All authors contributed to the article and approved the submitted version.

## Conflict of Interest

PV reports personal fees from Roche, outside the submitted work.

The remaining authors declare that the research was conducted in the absence of any commercial or financial relationships that could be construed as a potential conflict of interest.

## Publisher’s Note

All claims expressed in this article are solely those of the authors and do not necessarily represent those of their affiliated organizations, or those of the publisher, the editors and the reviewers. Any product that may be evaluated in this article, or claim that may be made by its manufacturer, is not guaranteed or endorsed by the publisher.
