# Corrigendum: IgG anti-spike antibodies and surrogate neutralizing antibody levels decline faster 3 to 10 months after BNT162b2 vaccination than after SARS-CoV-2 infection in healthcare workers

**DOI:** 10.3389/fimmu.2022.1098285

**Published:** 2022-11-25

**Authors:** Bram Decru, Jan Van Elslande, Sophie Steels, Gijs Van Pottelbergh, Lode Godderis, Bram Van Holm, Xavier Bossuyt, Johan Van Weyenbergh, Piet Maes, Pieter Vermeersch

**Affiliations:** ^1^ University Hospitals Leuven, Clinical Department of Laboratory Medicine and National Reference Center for Respiratory Pathogens, Leuven, Belgium; ^2^ Academic Centre of General Practice, KU Leuven, Leuven, Belgium; ^3^ Department of Public Health and Primary Care, KU Leuven, Leuven, Belgium; ^4^ Environment and Health, Department of Public Health and Primary Care, KU Leuven, Leuven, Belgium; ^5^ Group IDEWE, External Service for Prevention and Protection at Work, Heverlee, Belgium; ^6^ Laboratory of Clinical and Epidemiological Virology, Department of Microbiology, Immunology and Transplantation, Rega Institute, KU Leuven, Leuven, Belgium; ^7^ Department of Cardiovascular Sciences, KU Leuven, Leuven, Belgium

**Keywords:** SARS-CoV-2, COVD-19 serological testing, neutralizing antibodies, vaccination, spike, IgG, immunity, COVID-19

In the published article, there was an error regarding the equal contributions for Bram Decru and Jan Van Elslande. These authors have contributed equally to this work and share first authorship.

Bram Decru^1†^, Jan Van Elslande^1†^, Sophie Steels^1^, Gijs Van Pottelbergh^2,3^, Lode Godderis^2,3,4,5^, Bram Van Holm^6^, Xavier Bossuyt^1^, Johan Van Weyenbergh^6‡^, Piet Maes^6‡^ and Pieter Vermeersch^1,7*‡^

†These authors contributed equally to this work and share first authorship

‡These authors have contributed equally to this work and share senior authorship

The authors apologize for this error and state that this does not change the scientific conclusions of the article in any way. The original article has been updated.

## Publisher’s note

All claims expressed in this article are solely those of the authors and do not necessarily represent those of their affiliated organizations, or those of the publisher, the editors and the reviewers. Any product that may be evaluated in this article, or claim that may be made by its manufacturer, is not guaranteed or endorsed by the publisher.

