# Supplementary material for: IgG Anti-Spike Antibodies and Surrogate Neutralizing Antibody Levels Decline Faster 3 to 10 Months After BNT162b2 Vaccination Than After SARS-CoV-2 Infection in Healthcare Workers
Source: Front Immunol. 2022 Jun 15;13:909910. doi: 10.3389/fimmu.2022.909910 (PMC9241488; doi:10.3389/fimmu.2022.909910)
Supplement: Supplementary file 1 [file DataSheet_1.docx]

Supplementary Material

# Supplementary Figures and Tables

## Supplementary Figures


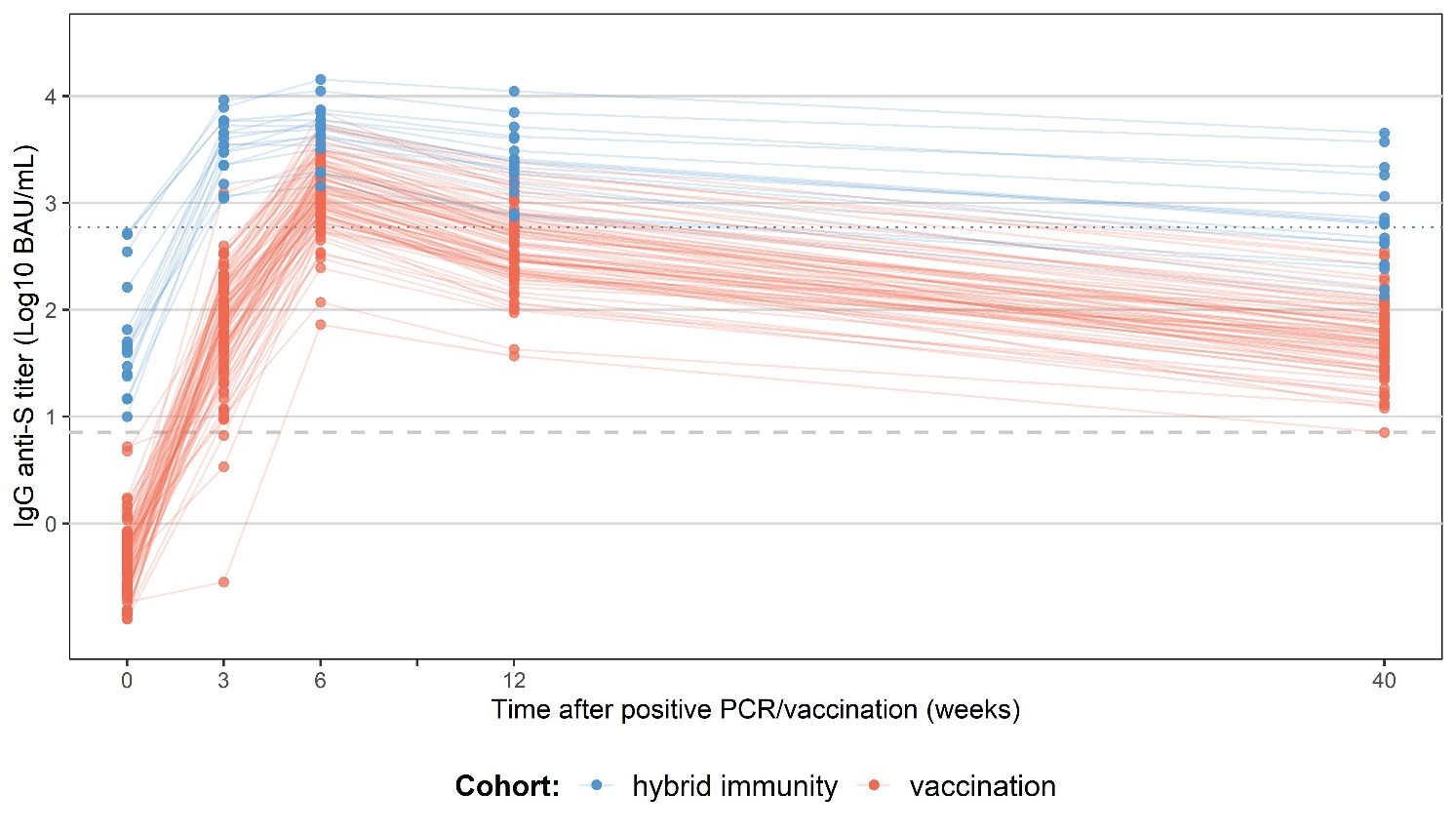


**Supplementary Figure 1.** Supplemental figure 1: Dynamic evolution of individual IgG anti-S antibody levels. The manufacturer’s cut-off for positivity 7,1 BAU/mL (0,85 on the log_10_ scale) and the cut-off for high probability of neutralizing antibody titer at 591 BAU/mL (2,8 on the log_10_ scale) are depicted as dotted and short dashed horizontal grey lines, respectively*.*

.
